# Comprehensive analysis of the expression, prognostic, and immune infiltration for COL4s in stomach adenocarcinoma

**DOI:** 10.1186/s12920-024-01934-3

**Published:** 2024-06-21

**Authors:** Ying Xu, Hangbin Jin, Yan Chen, Zhen Yang, Dongchao Xu, Xiaofeng Zhang, Jianfeng Yang, Yu Wang

**Affiliations:** 1 Department of Gastroenterology, Affiliated Hangzhou First People’s Hospital, Westlake University School of Medicine, Hangzhou, China; 2 Hangzhou Institute of Digestive Diseases, Hangzhou, China; 3 Key Laboratory of Integrated Traditional Chinese and Western Medicine for Biliary and Pancreatic Diseases of Zhejiang Province, Hangzhou, China; 4 Key Laboratory of Clinical Cancer Pharmacology and Toxicology Research of Zhejiang Province, Hangzhou, Zhejiang China; 5 School of Life Sciences, Zhejiang Chinese Medical University, Hangzhou, China

**Keywords:** Stomach adenocarcinoma, COL4, Prognosis, Immune infiltration, Biomarker

## Abstract

**Background:**

Collagen (COL) genes, play a key role in tumor invasion and metastasis, are involved in tumor extracellular matrix (ECM)-receptor interactions and focal adhesion pathways. However, studies focusing on the diagnostic value of the COL4 family in stomach adenocarcinoma (STAD) are currently lacking.

**Methods:**

The TCGA database was employed to retrieve the clinical features and RNA sequencing expression profiles of patients with STAD. We conducted an investigation to examine the expression disparities between STAD and adjacent normal tissues. Kaplan-Meier survival analysis was utilized to assess their prognostic significance, while Spearman correlation analysis was employed to determine their association with immune checkpoint genes and immunomodulatory molecules. Furthermore, GO and KEGG analyses were performed on the COL4s-related genes, revealing potential biological pathways through gene set enrichment analysis (GSEA). Subsequently, we explored the extent of immune infiltration of the COL4 family in STAD using the TIMER database. Lastly, the expression levels of the COL4 family in STAD were further validated through quantitative PCR (qPCR) and western blot techniques.

**Results:**

The expression levels of COL4A1/2 were significantly upregulated, while COL4A5/6 were conspicuously downregulated in STAD. The survival analysis revealed that the upregulated COL4s indicated poorer overall survival, first progression and post-progression survival outcomes. Additionally, our findings demonstrated a positive correlation between the expressions of COL4A1/2/3/4 and the infiltration of immune cells, including CD8 + T cells, dendritic cells, macrophages, neutrophils and CD4 + T cells. Further correlation analysis uncovered a favorable association between the expression of COL4A1/2/3/4 and various crucial immunomodulatory molecules, immunological checkpoint molecules, and chemokines. Quantitative PCR analysis confirmed that the expression patterns of COL4A1/3/4/6 genes aligned with the finding from the TCGA database. However, gastric cancer cells exhibited downregulation of COL4A2. Consistently, the protein level of COL4A1 was elevated, whereas the protein level of COL4A2 was reduced in the gastric cancer cell lines.

**Conclusion:**

COL4s could potentially serve as biomarkers for diagnosing and predicting the prognosis of STAD.

**Supplementary Information:**

The online version contains supplementary material available at 10.1186/s12920-024-01934-3.

## Introduction

According to the 2020 Cancer Study, gastric cancer (GC) ranks as the fifth most prevalent cancer globally (5.6%), and it has the fourth-highest mortality rate (7.7%) of all malignancies [1]. Stomach adenocarcinoma (STAD), specifically, comprises approximately 90% of GC cases. However, due to the absence of distinct clinical symptoms, a staggering 80-90% of STAD patients are generally not diagnosed until the disease has progressed to a more advanced stage [2]. In the recent decades, the advancement of bioinformatics, has enabled the discovery of numerous potential key biomarkers for cancer diagnosis, prognostic evaluation, and novel therapeutic targets through the utilization of high-throughput platforms [3].

Collagen (COL), the primary constituent of the tumor extracellular matrix (ECM), holds a pivotal position in cancer biology [4]. Elevated COL cross-linking and deposition have been implicated in integrin signal transduction, a process that fuels tumor growth [5]. COL genes, involved in tumor ECM-receptor interactions and focal adhesion pathways, play a crucial role in tumor invasion and metastasis [6]. Previous studies have shown that the upregulation of COL1A1 [7], COL10A1 [8], COL1A2 and COL6A3 [9] is intimately associated with the invasion and metastasis of GC cells. Compared to normal tissue, the expression of COL10A1 was significantly upregulated in the colon, lung and GC [10, 11]. Furthermore, the high expression of COL3A1 and COL5A1 serves as a marker for GC progression and prognosis [12]. Guo et al. have corroborated that the overexpression of COL1A1 can activate the TGF-β signaling pathway, thereby potentiating tumor cell proliferation and migration [13]. In addition, COL1A1/2 stand out as novel biomarkers for STAD due to their common overexpressed and correlation with invasion and metastasis [14]. In summary, studies have amply demonstrated that the COL family plays a vital role in the progression of various cancers.

The COL4 family, consisting of six genes-COL4A1, COL4A2, COL4A3, COL4A4, COL4A5, and COL4A6, is a group of subtypes in the COL family. Tu et al. discovered that clear cell renal cell carcinoma (ccRCC) tissue exhibited significantly diminished expression of COL4A3/4/5/6 in comparison to normal renal tissue, while showing markedly elevated expression of COL4A1/2 [15]. Patients with negative COL4A2 and COL4A6 in extrahepatic bile duct cancer exhibited notably poorer prognoses than those with positive COL4A2 and COL4A6 [16]. The migratory and proliferative capacities of triple-negative breast cancer cells are substantially inhibited by COL4A2 siRNA [17]. Upregulation of COL4A1 promotes the proliferation and migration of invasive breast carcinoma [18]. However, studies focusing on the diagnostic potential of the COL4 family genes in STAD is currently deficient.

**Fig. 1:**
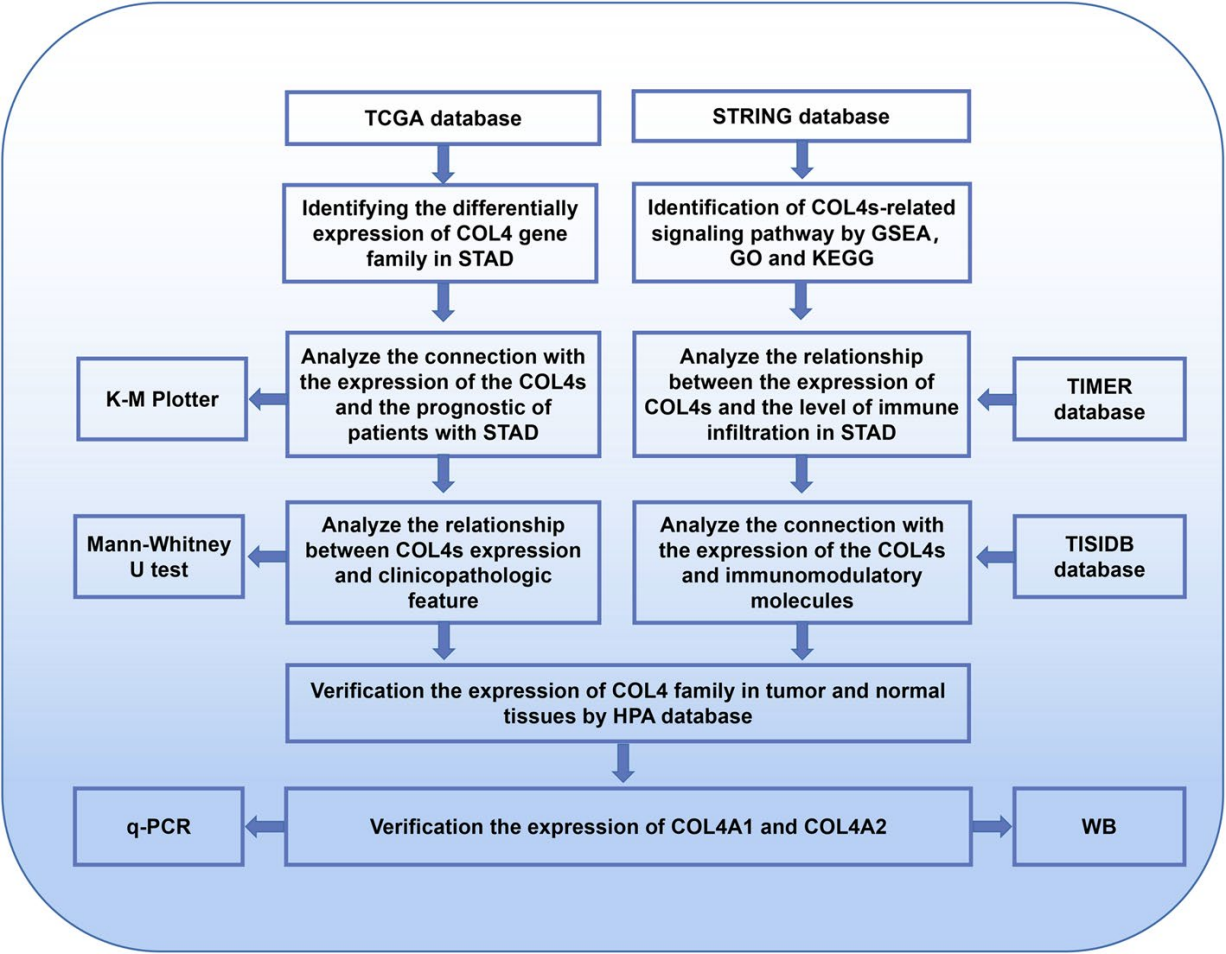
Flow chart of this study

In this study, we utilized the Cancer Genome Atlas (TCGA) database to assess the expression level and prognostic value of the COL4 family in STAD. Subsequently, we identified the pertinent interacting proteins and delved into the biological functions and mechanisms of COL4s. We further explored the associations between COL4s and immune infiltration by leveraging the Tumor Immunization Estimation Resource (TIMER) database and Tumor-Immune System Interaction Database (TISIDB). In addition, in vitro experiments were performed to validate the expression levels of COL4s, with the aim of offering a novel avenue for the diagnosis and treatment of STAD. Figure 1 depicts the complete flowchart of the study.

## Materials and methods

### Source of data and reagents

The gene expression matrix data were extracted from the Cancer Genome Atlas-Stomach adenocarcinoma (TCGA-STAD) database (https://tcga-data.nci.nih.gov/tcga/). By computing z-scores, the data were pre-processed and standardized.

Gastric cancer cell lines (HGC-27 and MKN-45) and normal gastric epithelial cells (GES-1) had been bought from Procell (China). Fetal bovine serum (FBS) and penicillin-streptomycin (PS) were obtained from Gibco (USA) and Invitrogen (USA). RPMI-1640 medium was obtained from Gibco (USA). At 37℃ in a humidified atmosphere containing 5% CO_2_, cells were grown in RPMI-1640 with 10% FBS and 1% PS.

### Differential analysis

Using TCGA data, the expression level of the COL4 family of genes was compared between cancers and tumor-adjacent normal tissues. This dataset includes grouped samples (375 tumor tissues and 32 tumor-adjacent normal tissues) and paired samples (consisting of 27 matched tumor/non-tumor tissues). Wilcoxon rank sum test was used for differential expression analysis of group samples, while Wilcoxon signed rank test was used for differential expression analysis of matched samples.

### Survival analysis and the relationship between the COL4A family and clinicopathological features

To explore the diagnostic values of the COL4A family, Kaplan-Meier survival analysis (http://kmplot.com/analysis/) was used to estimate survival functions [including overall survival (OS), first progression (FP), and post-progression survival (PPS)] and the log-rank test was applied to group comparisons. Relationship between the expression of COL4 family and clinicopathological features, including histological grade, pathological stage and T-stage, were analyzed by Mann-Whitney U-test and visualized using the ‘ggplot2’ package of R software (vision 3.3.3).

### Protein-Protein Interaction (PPI) network construction and functional enrichment analysis

Differential expression analysis was performed using the ‘DESeq2’ package of R software. The absolute value of log2 fold change (FC) > 1 and adjusted *P* value < 0.05 were the screening requirements. To explore interactively on COL4, the PPI network was constructed using the STRING database (https://cn.string-db.org/). The confidence score was set at 0.4, and other parameters were kept as default values. To create a network map, the interaction data were loaded into the Cytoscape program (version 3.9.1).

The ‘clusterProfiler’ of R was used to perform gene ontology (GO) and Kyoto Encyclopedia of Genes and Genomes (KEGG) analyses to find the probable biological functions and pathways for the COL4 family. Finally, gene set enrichment analysis (GSEA) (http://software.broadinstitute.org/gsea/index.jsp) was used to investigate the pathways associated with COL4s-related proteins. Enrichment analysis was performed using the values of the provided molecules to calculate the z-score value corresponding to each enriched entry via the ‘clusterProfiler’ package of R, and visualized with the ‘ggplot’ package of R.

### Correlation analysis of immune cell infiltration, immune checkpoints, and immunomodulatory molecules

TIMER database (https://cistrome.shinyapps.io/timer/) was used to assess the correlation of the COL4 family with infiltration of six immune cell types (including B cells, CD8 + T cells, CD4 + T cells, macrophages, neutrophils and dendritic cells) in STAD. To explore the correlation between the COL4 family and immunomodulators, TISIDB database (http://cis.hku.hk/TISIDB/) was used to obtain the Spearman correlation coefficient and *P*-value, and the ‘ggplot2’ package of R was used for visualized the result.

### Verification by quantitative polymerase chain reaction (qPCR)

Cell lines were pretreated and RNA was extracted. cDNA was synthesized using 1 µg of RNA using the Prime Script RT kit (TaKaRa, Dalian, China). PCR primers (Table S1) were synthesized and purchased through Sangon Biotech (Shanghai, China) and then performed using a 7500 real-time fluorescence quantitative PCR system.

### Verification by western blot

Human GC cell lines and regular human gastric mucosal epithelial cell line were subjected to total protein extraction. Proteins were separated on 10% SDS-PAGE and then electrophoretically transferred to PVDF membranes at 300 mA constant current. 5% skim milk was used to seal the membranes and then the membranes were incubated with COL4A1 (HA500197, Huabio, China) and COL4A2 (A7657, Abclonal, China) at 4℃ overnight. The membranes were then washed 3 times with 0.1% TBST buffer and incubated with horseradish peroxidase (HRP)-coupled secondary antibody (A0208, Beyotime, China) for 1 h at room temperature, followed by washing TBST buffer 3 times. GAPDH was used as a loading control.

### Immunohistochemical validation of COL4 family expression in STAD and normal tissues

Immunohistochemical maps of the COL4 family were obtained from the Human Protein Atlas (HPA) database (https://www.proteinatlas.org/) for further validation of protein expression levels.

### Statistical analysis

R software (version 3.3.3) and SPSS 20.0 were utilized for statistical analysis. Prior to analysis, data underwent assessments for homogeneity of variances and normality. Shapiro-Wilk normality test was used for normality tests, while Levene’s test was used to test the homogeneity of variances. Student’s t-test was applied for statistical analysis when the data were normally distributed and variances were equal. In cases where the data did not meet the assumptions of equal variances, Welch’s t-test was employed. Alternatively, the Wilcoxon rank sum test was utilized if the data did not satisfy the assumptions of parametric tests. Spearman correlation analysis was used to conduct correlation analysis. For qPCR, student t-test was used to analyze continuous variables. For western blot, band intensities were quantified using ImageJ software (version 1.49) and used to calculate the relative protein levels normalized to GAPDH. Statistical significance was indicated by *P* < 0.05.

## Results

### Expression level of COL4s was abnormal in STAD

**Fig. 2:**
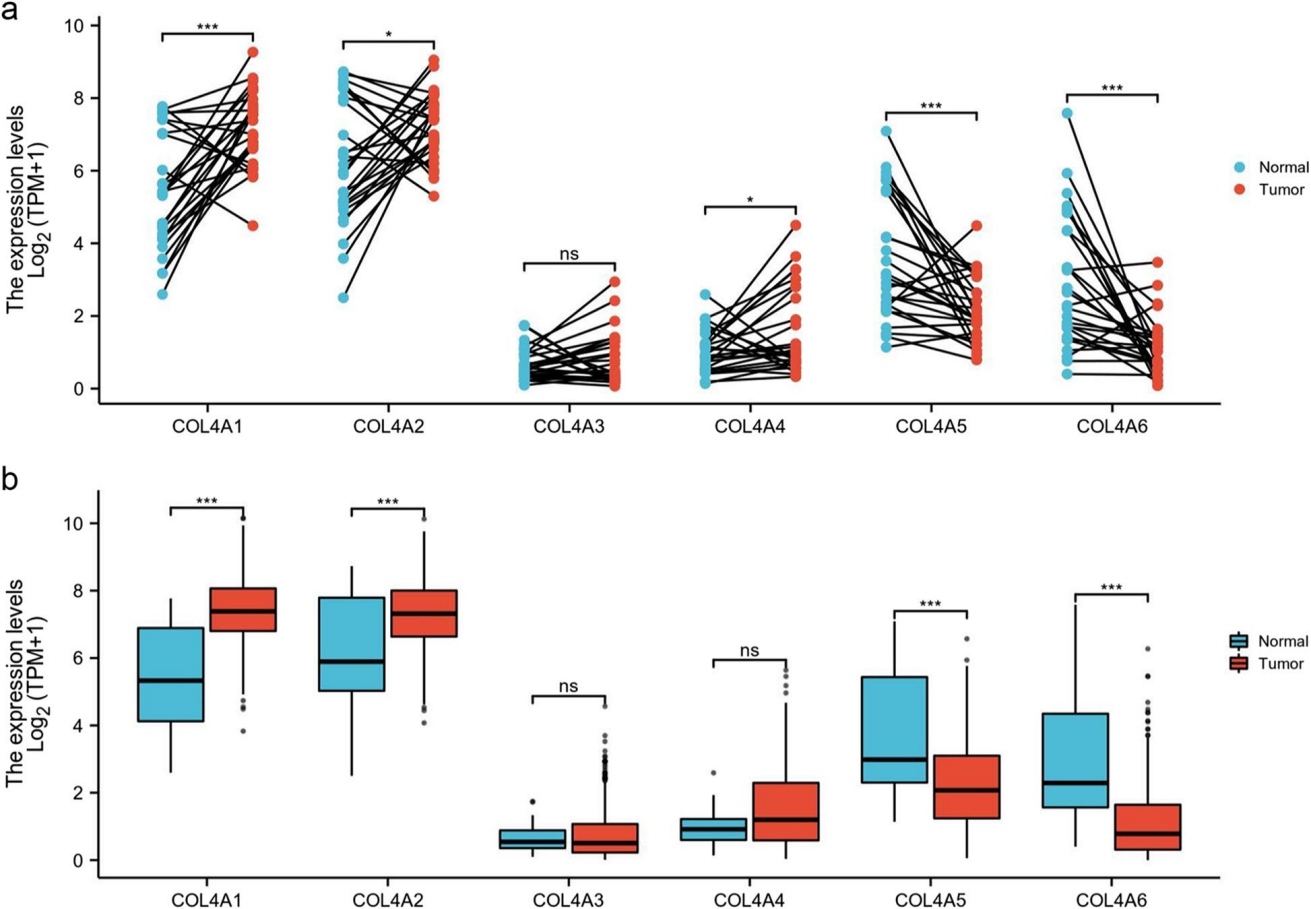
Differential expression of COL4 family in STAD. **a** mRNA expression of COL4 family in STAD paired samples. **b** mRNA expression of COL4 family in STAD grouped samples. *P < 0.05; ***P < 0.001; ns *P* > 0.05

In our initial investigation, we assessed the mRNA expression levels of the COL4 family in both STAD tumors and normal tissues. Paired sample analysis revealed a significant increase in the expression levels of COL4A1, COL4A2, and COL4A4, whereas COL4A5 and COL4A6 showed a significant decrease in STAD tissues compared to normal adjacent tissues (*n* = 27) (Fig. 2a). Similarly, analysis of the grouped samples demonstrated a notable upregulation of COL4A1 and COL4A2 expression, along with a significant downregulation of COL4A5 and COL4A6 in STAD tissues (*n* = 375) compared to normal adjacent tissues (*n* = 32) (Fig. 2b).

### COL4 family expression is related to the prognosis and clinicopathological features of STAD patients

**Fig. 3:**
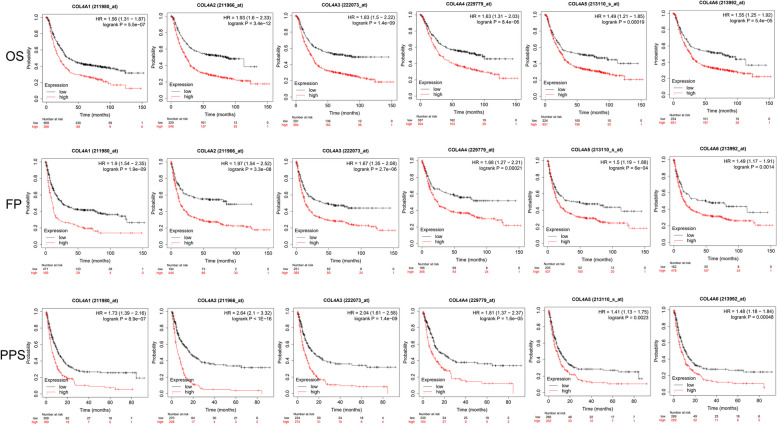
mRNA expression correlates with prognosis in STAD. Kaplan-Meier plot showing the relationship between OS, FP, PPS and COL4s expression in patients. The black curve represents the low expression group and the red curve represents the high expression group. The number of patients in the low and high expression groups is shown below the curve. HR: Hazard Ratio

**Fig. 4:**
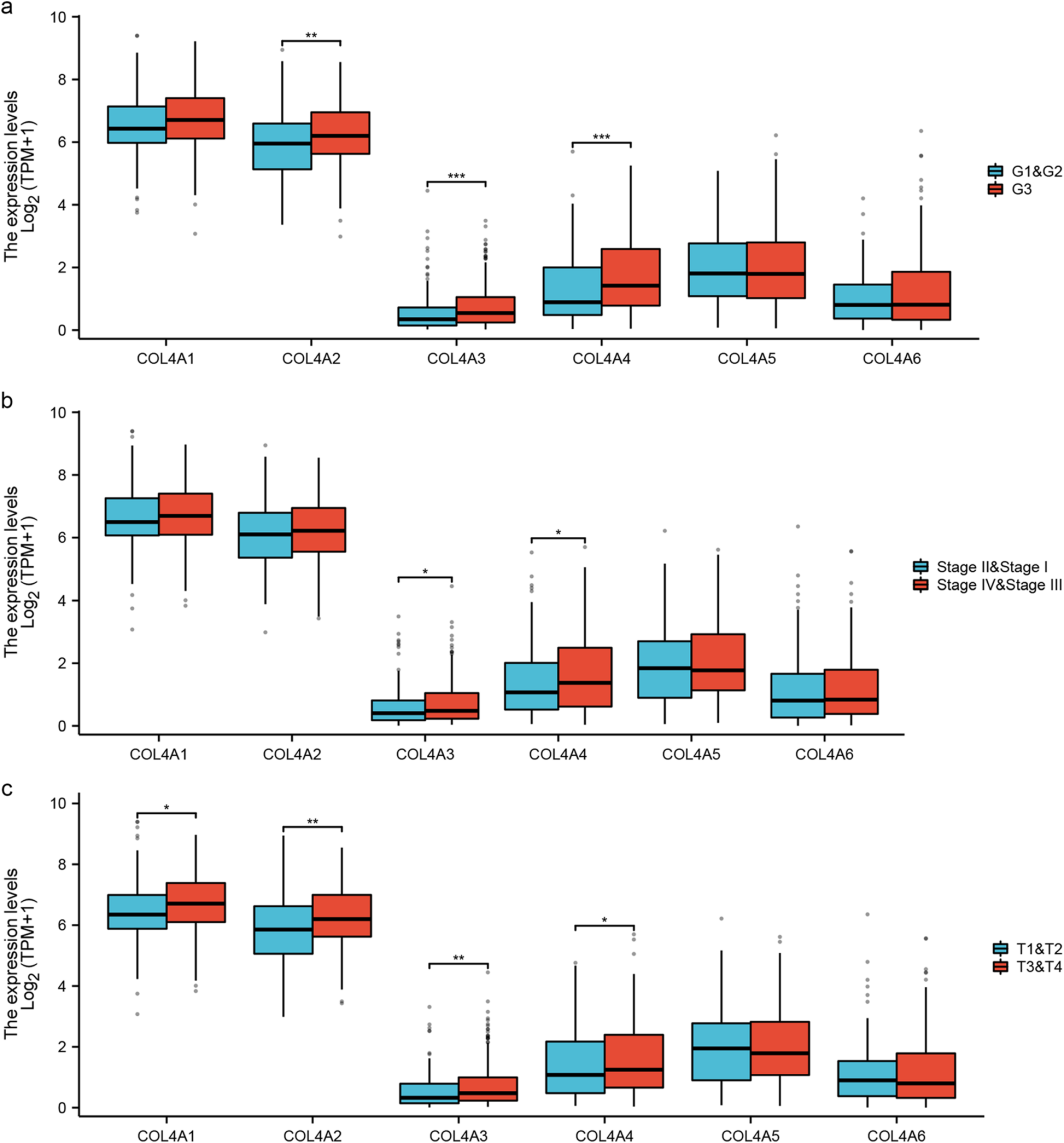
Relationship between COL4s and clinicopathological characteristics in STAD. Relationship between each member of COL4 gene family and histological grading (**a**), pathological stage (**b**), and T stage (**c**) in STAD patients. ^*^P < 0.05; ^**^P < 0.01; ^***^P < 0.001; ns *P* > 0.05

We also tested whether COL4 family mRNA expression correlates with STAD prognosis. Results showed that the high expression of COL4A1/2/3/4/5/6 mRNA was significantly associated with poor OS, FP and PPS (*P* all < 0.01, Fig. 3). Additionally, we analyzed the relationship between COL4s gene expression levels and various clinical characteristics. The mRNA levels of COL4A2, COL4A3, and COL4A4 were significantly higher in the histological G3 stage compared to the G1 and G2 stages (*P* < 0.05, Fig. 4a). Moreover, COL4A3 and COL4A4 mRNA expressions were significantly higher in pathological stage IV and stage III compared to stage I and stage II (*P* < 0.05, Fig. 4b). Compared with stages T1 and T2, mRNA expressions of COL4A1/2/3/4 were significantly higher in stages T3 and T4 (*P* < 0.05, Fig. 4c).

### Protein interaction (PPI) network and functional enrichment analysis

**Fig. 5:**
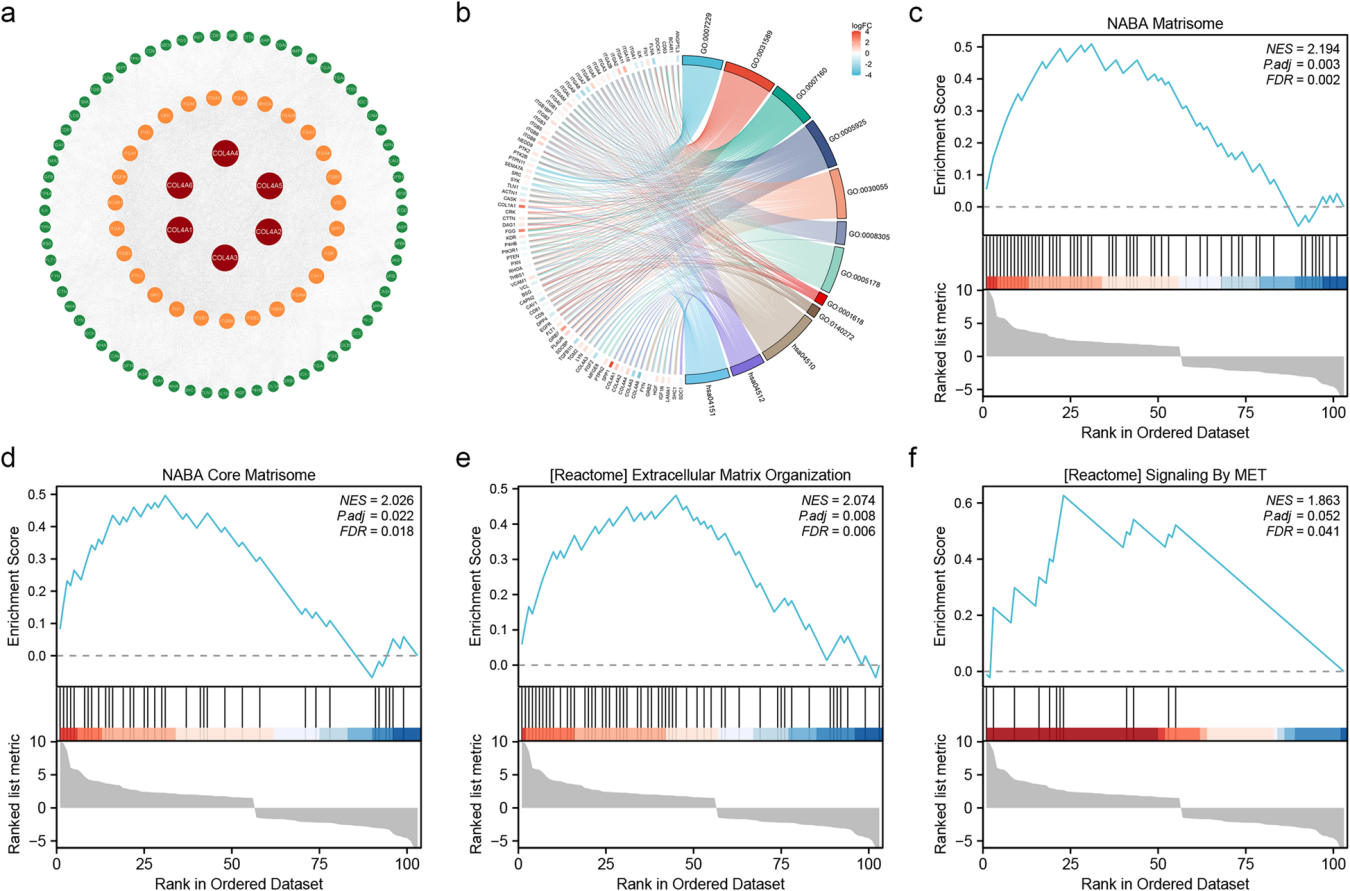
Functional enrichment analysis of COL4 family. **a** PPI networks present proteins that interact with COL4s. The red circles are the COL4s, and the orange and green circles are genes that interact with this family. **b** GO enrichment analysis and KEGG pathway enrichment analysis of COL4 family. **c-f** Enrichment maps from GSEA. NES: normalized enrichment score, *P*. adj: adjusted *p *value; FDR: false discovery rate

To analyze interaction networks among the COL4 family, we performed PPI analysis using the STRING database and visualized by Cytoscape (Fig. 5a). As shown in the figure (100 nodes and 3502 edges), genes related to the COL4 family include integrin-associated genes (ITGAM, ITGB2, ITGB1, ITGB6, ITGB3, ITGAV, ITGA1, ITGA5, ITGA2, ITGA6, ITGA2B, ITGA3, ITGA4, ITGB5, SPP1, CAV1), cytoskeletal genes (PXN, VCL), fibronectin genes (FN1), ECM-associated genes (PTK2, THBS1, BCAR1) and endothelial growth factor genes (EGFR, KDR). All these genes are closely related to the ECM.

In order to delve into the potential biological processes linked with the COL4 family, we conducted GO and KEGG enrichment analyses utilizing the COL4 family and their associated genes acquired from the STRING. Our findings unveiled that the COL4 family and their related genes were predominantly enriched in several key pathways and functions. These encompassed the integrin-mediated signaling pathway, cell-substrate adhesion, cell-matrix adhesion, focal adhesion, cell-substrate junction, integrin complex, integrin binding, virus receptor activity, exogenous protein binding, ECM-receptor interaction, and the PI3K-Akt signaling pathway (Fig. 5b).

The potential biological pathways were explored through gene set enrichment analysis (GSEA). Significantly enriched signaling pathways were displayed in supplementary Table 2 (Table S2). Pathway enrichment analysis indicated that COL4s-related genes were mainly involved in the terms naba matrisome (Fig. 5c); naba core matrisom (Fig. 5d); extracellular matrix organization (Fig. 5e); signaling by met (Fig. 5f).

### COL4 family expression is related to immune infiltration levels in STAD

**Fig. 6:**
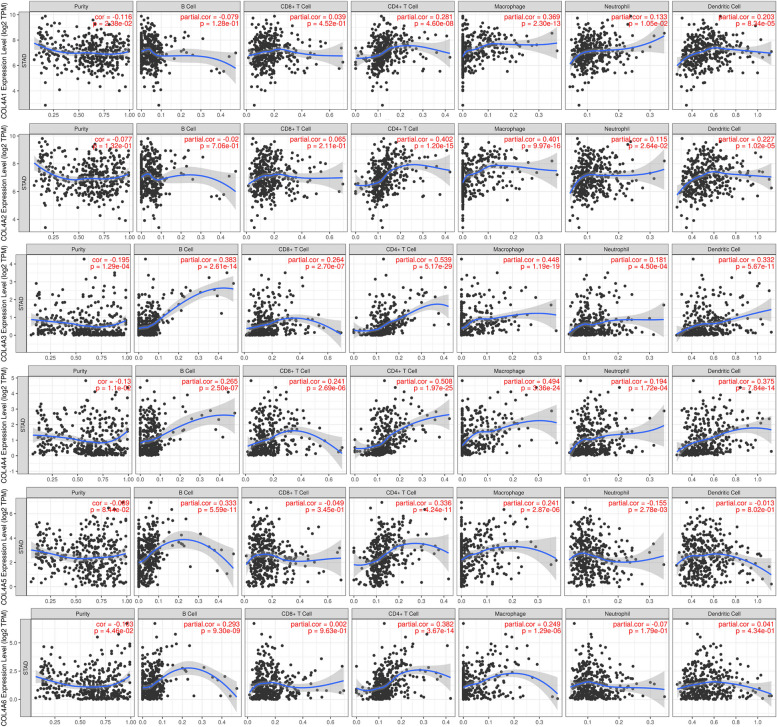
Correlation analysis between COL4s and immune cell infiltration

Furthermore, the relationship between COL4As expression levels and immune cell infiltration (including CD8 + T cells, B cells, dendritic cells, macrophages, neutrophils, and CD4 + T cells) was investigated. Results showed that COL4A1 was negatively correlated with tumor purity, and positively correlated with CD8 + T cell, dendritic cell, macrophage, neutrophil, and CD4 + T-cell infiltration. In addition, the association between the level of immune cell infiltration and COL4A2 expression was also significant. COL4A2 did not correlate with tumor purity and B-cell infiltration but positively correlated with CD8 + T-cell, dendritic cell, macrophage, neutrophil, and CD4 + T-cell infiltration. COL4A3/4 was negatively correlated with tumor purity while positively correlated with CD8 + T cells, B cells, dendritic cells, macrophages, neutrophils, and CD4 + T cell infiltration. On the other hand, COL4A5 showed a positive correlation with macrophages and CD4 + T cells but not with other immune cell types. Similarly, COL4A6 was positively correlated with macrophage, neutrophil and CD4 + T cell immune infiltration, however, negatively correlated with tumor purity (Fig. 6).

### COL4 family expression is related to immunomodulatory molecules and immune checkpoints in STAD

**Fig. 7:**
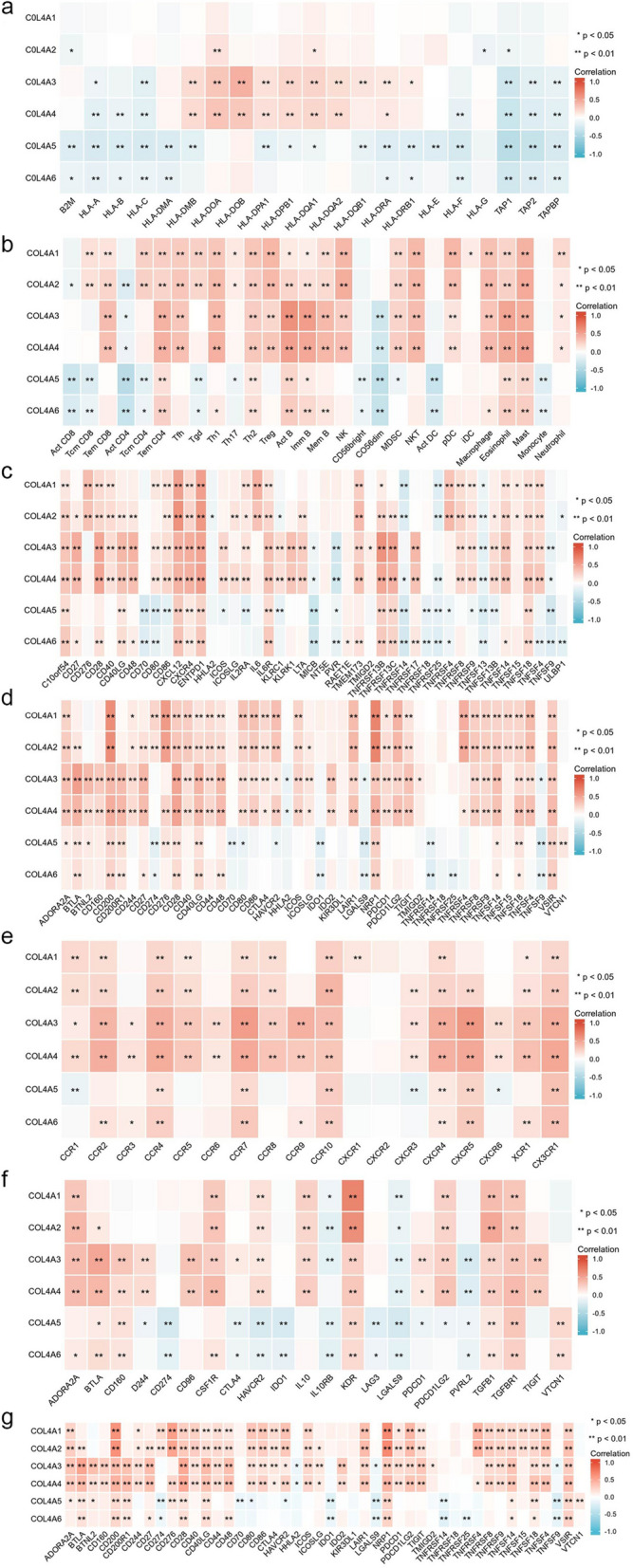
Correlation of COL4 family expression with immune checkpoints and immunomodulatory molecules. Correlation between COL4 family genes and immunostimulants (**a**), immunosuppressants (**b**), MHC molecules (**c**), lymphocytes (**d**), receptors (**e**), chemokines (**f**), and immune checkpoints (**g**). Red color indicates positive correlation and blue color indicates negative correlation. Color intensity indicates the strength of the correlation. ^*^P < 0.05; ^**^P < 0.01

Correlation between the COL4 family and immunostimulants, immunosuppressants, MHC molecules, lymphocytes, receptors, and chemokines were assessed using Spearman correlation analysis. From the heat map (Fig. 7), we can visualize that almost all immune-related genes were significantly positively correlated with COL4A3/4. Whereas COL4A1 was positively associated with most of the immunosuppressants (21/30), MHC molecules (21/45), lymphocytes (28/46), receptors (11/18) and a few chemokines (8/22) but not associated with immunostimulants. Similarly, COL4A2 was positively associated with most of the immunosuppressants (22/30), MHC molecules (27/45), lymphocytes (31/45) and receptors (12/18). In addition, COL4A2 was also positively associated with a few chemokines (9/22) and one immunostimulant. Interestingly, COL4A5/6 were negatively associated with immunostimulants and significantly positively associated with some immunosuppressants (6/30, 9/30), MHC molecules (9/45, 16/45), lymphocytes (13/46, 11/46), receptors (6/18, 10/18), and chemokines (6/22, 8/22). In addition, we explored the correlation between the COL4 family and 46 immune checkpoints. Results indicated that the expression of key immune checkpoints such as CD200, CD28, CD40LG, NRP1, TNFSF14, TNFSF18, and VSIR were positive correlation with the COL4 family.

### Immunohistochemical results validate COL4 family expression in STAD and normal tissues

**Fig. 8:**
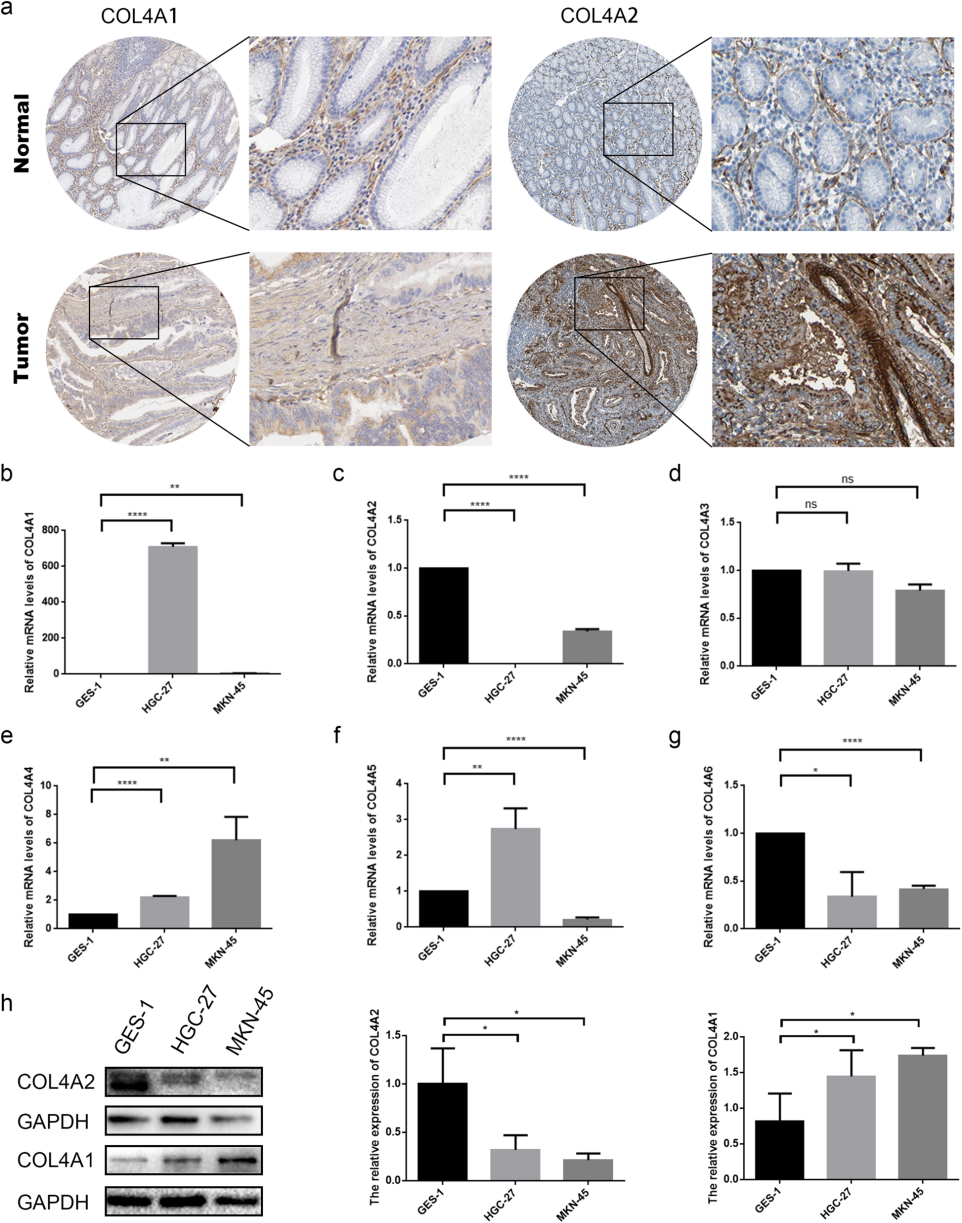
Immunohistochemistry and experimental verification. **a** The protein expression of COL4A1 and COL4A2 in immunohistochemical images between normal and tumor tissues. **b-g** The mRNA expression levels of COL4A1 (**b**), COL4A2 (**c**), COL4A3 (**d**), COL4A4 (**e**), COL4A5 (**f**) and COL4A6 (**g**) in GES-1, HGC-27 and MKN-45 were measured by qPCR. The results were normalized to the reference gene GAPDH. **h** Western blot protein detection of the COL4A1 and COL4A2 expression levels in cell lines. Images of blots intercepted from different parts of the same print, or from different blots separated by dividing lines. ^*^P < 0.05; ^**^P < 0.01; ^****^P < 0.0001; ns *P* > 0.05

Immunohistochemical staining data of COL4 family members were used to verify their expression in STAD and normal tissues. The results demonstrated that COL4A1 and COL4A2 exhibited higher expression levels in tumor samples compared to normal tissues (Fig. 8a).

### In vitro experiments verification of COL4s in STAD by qPCR and western blot

PCR experiments were conducted to validate the findings from the previous analysis. Compared to GES-1, COL4A1 expression was seven hundred-fold higher in HGC-27 and four-fold higher in MKN-45 (Fig. 8b). Conversely, COL4A3 expression had no significant difference between STAD cells and normal gastric mucosal epithelial cells (Fig. 8d). Compared to GES-1, COL4A4 expression was significantly higher in both HGC-27 and MKN-45 (Fig. 8e), while COL4A6 was significantly lower in HGC-27 and MKN-45 (Fig. 8g). These results were consistent with the TCGA database. Differently, the expression of COL4A2 was significantly reduced in HGC-27 and MKN-45 (Fig. 8c). Interestingly, compared with the expression of COL4A5 in GES-1, it was 2-fold highly expressed in HGC-27, while 5-fold lower in MKN-45 (Fig. 8f). These results need to be explored further in depth.

To validate the mRNA expression pattern, we compared the protein expression levels of COL4A1 and COL4A2 between tumor cells and normal cells. As depicted in Fig. 8h, the protein expression of COL4A1 and COL4A2 corroborated with the qPCR results, providing further validation of the previously observed mRNA expression findings.

## Discussion

STAD stands out with the highest incidence and mortality rate among digestive system cancers in Eastern Asia [19]. The absence of reliable diagnostic options, trustworthy biomarkers, and particular symptoms during the early stages often leads to late-stage diagnoses for many patients, profoundly impacting the prognosis of individuals with STAD [20, 21]. Previous investigations have explored the impact of the COL4 family on cancer development, revealing their involvement in the proliferation, metastasis and invasion of various cancers [16–18]. Among them, overexpression of COL4A1 has been linked to the proliferation of breast cancer cells [18] and has been identified as a prognostic biomarker for intrahepatic cholangiocarcinoma [22]. Huang et al. discovered that COL4A1 was upregulated in GC cells resistant to trastuzumab, suggesting a potential role for COL4A1 in conferring resistance to trastuzumab in GC [23]. In patients with hepatocarcinogenesis, overexpression of COL4A2 was positively correlated with a shorter progression-free survival [24]. Patients with low COL4A3 expression in non-small cell lung cancer exhibited considerably longer median survival times compared to those with high COL4A3 expression [25]. Knockdown of COL4A3 significantly increased the invasion and migration ability of nasopharyngeal carcinoma cancer cells [26]. The diminished expression of COL4A4 is associated with poorer prognosis, while the downregulation of COL4A6 promotes prostate cancer progression and invasion [15, 27].

In this study, analysis conducted on the TCGA database revealed a significant upregulation of COL4A1/2/4 expression in STAD tumor tissues. Conversely, the expression of COL4A5/6 was downregulated in tumor tissues and the expression of COL4A3 had no significant difference between tumor and normal tissues. In addition, data from the Kaplan-Meier plotter database indicated a notable association between high expression levels of COL4s and poor OS, FP and PPS. Our pathological characterization results underscored a significant correlation between elevated expression of COL4A1/2/3/4 and advanced G3 and T3 & T4 stages. Moreover, high expression of COL4A3/4 was associated with a worse pathological stage. According to the HPA database, expression of COL4A1/2 was high in STAD tissues but low in normal tissue samples. Prior research has also demonstrated a significant upregulation of COL4A1/2 in precancerous and hepatocellular carcinoma tissues [23]. Our in vitro experiments further supported these findings, demonstrating the upregulation of COL4A1 mRNA and protein expression levels in HGC-27 and MKN-45 cell lines. However, contrary to expectations, qPCR and western blot results revealed a reduction in the expression of COL4A2 in the HGC-27 and MKN-45 cell lines. This disparity may stem from inherent biological differences among cell lines originating from the same tumor, influenced by their distinct genetic backgrounds. Moreover, it is pertinent to acknowledge that primary and metastatic tumor tissues and cells can exhibit divergent biological characteristics even within the same patient. Hence, it is crucial to include a larger number of clinical samples to further validate the expression patterns of COL4A2.

QPCR results indicated that COL4A3 was not significantly different between GC cells and gastric epithelial cells, COL4A4 was highly expressed while COL4A5/6 were lowly expressed in GC cell lines. Previous research has reported the downregulation of COL4A5 and COL4A6 expression in colorectal cancer [28]. However, due to the lack of available antibodies, COL4A4/5/6 could not be validated at the protein level. Further studies are warranted to explore the potential roles of COL4A4/5/6 in STAD.

The PPI results underscore the close association between the COL4 family and the integrin family of protein-coding genes. KEGG and GSEA analysis showed that the COL4 family was enriched in integrin binding and adhesion-related pathways, such as integrin-mediated signaling, cell-substrate adhesion, and PI3K-Akt signaling pathways. Previous studies have demonstrated that COL4A1 can active the TGF-/PI3K/AKT pathway, which is related to the proliferation, migration, and invasion of STAD in vitro and in vivo [29, 30]. Moreover, bioinformatics analyses conducted by Liu et al. have elucidated that COL4A1/2 binds to integrin α-2 /β-1 accelerating the cell cycle and promoting hepatocarcinogenesis, where the key activation signal is the PI3K-Akt pathway [24]. Based on the results of our enrichment analysis, we speculate that COL4s affect the migration and progression of STAD by regulating the PI3K-Akt signaling pathway. In forthcoming experiments, we intend to manipulate the expression levels of COL4s through overexpression and knockdown techniques, subsequently evaluating the expression levels of Akt or PI3K, alongside assessing the migration and invasion capabilities of cancer cells. Moreover, there was a positive correlation between the expression of COL4A1/2/3/4 and immune cell infiltration, including CD8 + T cells, dendritic cells, macrophages, neutrophils, and CD4 + T cells, while the expression of COL4A5/6 was positively correlated with macrophages and CD4 + T cells. The expression of COL4A1 was significantly positively correlated with the markers of tumor immune cell infiltration (such as Treg, M2 and TAM) and immunosuppressive cytokines [31]. Our results suggested that COL4A1/2/3/4 may play a role in tumor immunosuppression and may have great potential as immune infiltration markers with important implications for tumor diagnosis and target development.

In summary, our study delved into the distinct expression patterns of COL4 family genes in tumor tissues relative to normal tissues and elucidated their correlation with clinical prognosis. Through a convergence of experimental and database analyses, we consistently observed a marked upregulation of COL4A1 expression in STAD, along with a close association with tumor-infiltrating immune cells. These findings suggest the potential utility of COL4A1 as a promising predictive marker for assessing both the prognosis and treatment efficacy in STAD.

It is crucial to recognize several limitations in our study. Firstly, a substantial portion of the data utilized in our research was derived from the TCGA database. The absence of certain clinical parameters may have affected the overall quality of our investigation. Secondly, although we conducted experimental verification on the differential expression of genes and proteins within the COL4 family, this verification was limited to the two cell lines, HGC-27 and MKN-45. It is crucial to acknowledge that there may be distinct expression profiles in other STAD cell lines, which remain unexplored. Additionally, we have not delved into their functional phenotypes specifically in the context of STAD. Future studies should prioritize conducting high-quality experimental investigations such as adding more cell lines and clinical samples, to further elucidate and validate our findings. Thirdly, the exploration of the underlying mechanisms in our study primarily relied on gene functional annotation and enrichment analysis, lacking in vivo or in vitro experimental verification. Nonetheless, our study contributes novel insights by revealing a significant increase in COL4A1 expression, along with a strong correlation with tumor-infiltrating immune cells in STAD. COL4A1 holds the potential as a prognostic marker for evaluating the efficacy of immunotherapy and monitoring the progression of STAD. These findings offer valuable references for the prognosis of immunotherapy and suggest new targets for the development of immunosuppressive agents.

## Data availability

These online databases can be accessed from the following addresses. TCGA-STAD database (https://tcga-data.nci.nih.gov/tcga/), GSEA database (http://software.broadinstitute.org/gsea/index.jsp), Kaplan-Meier Plotter (https://kmplot.com/analysis/index.php?p=service), STRING database (https://cn.string-db.org/), TIMER database (https://cistrome.shinyapps.io/timer/), TISIDB database (http://cis.hku.hk/TISIDB/), and HPA database (https://www.proteinatlas.org/).

### Supplementary Information

Supplementary Material 1.

## Abbreviations

COL: Collagen
STAD: Stomach Adenocarcinoma
ECM: Extracellular matrix
GSEA: Gene set enrichment analysis
qPCR: quantitative PCR
GC: Gastric cancer
ccRCC: clear cell renal cell carcinoma
TCGA: The Cancer Genome Atlas
TIMER: The Tumor Immunization Estimation Resource
FBS: Fetal bovine serum
PS: Penicillin-streptomycin
OS: Overall survival
FP: First progression
PPS: Post-progression survival
FC: Fold of change
GO: Gene ontology
KEGG: Kyoto Encyclopedia of Genes and Genomes
HRP: Horseradish peroxidase
HPA: Human Protein Atlas
TISIDB: Tumor-Immune System Interaction Database
