# Supplementary material for: Comprehensive analysis of the expression, prognostic, and immune infiltration for COL4s in stomach adenocarcinoma
Source: BMC Med Genomics. 2024 Jun 21;17:168. doi: 10.1186/s12920-024-01934-3 (PMC11191235; doi:10.1186/s12920-024-01934-3)
Supplement: Supplementary file 1 — Supplementary Material 1. [file 12920_2024_1934_MOESM1_ESM.docx]

Supplementary Material

# Supplementary Tables

## Supplementary table 1

**Table S1** Primer sequences used for q-PCR

| Gene | Forward primer (5’-3’) | Reverse primer (5’-3’) |
| --- | --- | --- |
| COL4A1  COL4A2  COL4A3  COL4A4  COL4A5  COL4A6  GAPDH | TGAGAAGGCAATGAACGC  CTGGGTGGCGGAGTTTGT  ACGAGCCCACGGACAAGA  AGGGTCTTGCCTTCCCGTAT  TGCCTTCGTCGCTTTAGT  GATGGCTGTAATGGAACTCA  TGCACCACCAACTGCTTA | GTGGAGCAAACAATATCAAACA  ACTTGTTGGCGTAGTAGTGGC  TGTTCATTGGCATCAGAGC  CAGCCAGTAGGATCTGTCGTTT  TGAATGGCTGGATGCTCT  CTGGAGCAAGGACAGGGT  GGATGCAGGGATGATGTTC |

## Supplementary table 2

**Table S2** GO and KEGG enrichment analysis results

| ID | Description | *p* value | *p*.adjust | q value |
| --- | --- | --- | --- | --- |
| GO:0007229 | integrin-mediated signaling pathway | 2.07077E-59 | 6.20195E-56 | 3.33284E-56 |
| GO:0031589 | cell-substrate adhesion | 5.06577E-54 | 7.58599E-51 | 4.07661E-51 |
| GO:0007160 | cell-matrix adhesion | 2.65845E-52 | 2.65402E-49 | 1.42624E-49 |
| GO:0005925 | focal adhesion | 1.40687E-48 | 3.54532E-46 | 2.11771E-46 |
| GO:0030055 | cell-substrate junction | 3.76563E-48 | 4.74469E-46 | 2.83413E-46 |
| GO:0008305 | integrin complex | 6.0809E-45 | 5.10796E-43 | 3.05112E-43 |
| GO:0005178 | integrin binding | 4.50587E-62 | 1.36979E-59 | 9.10661E-60 |
| GO:0001618 | virus receptor activity | 7.78395E-15 | 9.30262E-13 | 6.18457E-13 |
| GO:0140272 | exogenous protein binding | 9.18022E-15 | 9.30262E-13 | 6.18457E-13 |
| hsa04510 | Focal adhesion | 4.42951E-64 | 7.92882E-62 | 3.54361E-62 |
| hsa04512 | ECM-receptor interaction | 2.27033E-40 | 2.03194E-38 | 9.08131E-39 |
| hsa04151 | PI3K-Akt signaling pathway | 2.96128E-31 | 1.76689E-29 | 7.89673E-30 |

GO, Gene Ontology; KEGG, Kyoto encyclopedia of genes and genomes.

## Supplementary table 3

**Table 3** Gene sets enriched for related genes

| **ID** | **NES** | ***p* value** | ***p*.adjust** | **q value** |
| --- | --- | --- | --- | --- |
| NABA_MATRISOME | 2.194421958 | 6.50891E-05 | 0.002863918 | 0.002260988 |
| REACTOME_EXTRACELLULAR_MATRIX_ORGANIZATION | 2.073750527 | 0.000353964 | 0.007787203 | 0.006147792 |
| NABA_CORE_MATRISOME | 2.025685226 | 0.001531196 | 0.022457544 | 0.01772964 |
| REACTOME_SIGNALING_BY_MET | 1.863313313 | 0.007132002 | 0.052301351 | 0.04129054 |
| REACTOME_MET_ACTIVATES_PTK2_SIGNALING | 1.863313313 | 0.007132002 | 0.052301351 | 0.04129054 |
| REACTOME_MET_PROMOTES_CELL_MOTILITY | 1.863313313 | 0.007132002 | 0.052301351 | 0.04129054 |
| NABA_ECM_GLYCOPROTEINS | 1.870168286 | 0.014148815 | 0.088935408 | 0.070212164 |
| REACTOME_ECM_PROTEOGLYCANS | 1.636282125 | 0.022013925 | 0.121076586 | 0.095586778 |
| REACTOME_ASSEMBLY_OF_COLLAGEN_FIBRILS_AND_OTHER_MULTIMERIC_STRUCTURES | 1.621743543 | 0.02575393 | 0.1259081 | 0.099401132 |
| REACTOME_RNA_POLYMERASE_II_TRANSCRIPTION | 1.714506927 | 0.037339302 | 0.161490683 | 0.127492645 |
| PID_SYNDECAN_1_PATHWAY | 1.590769824 | 0.040372671 | 0.161490683 | 0.127492645 |
| REACTOME_INTEGRIN_CELL_SURFACE_INTERACTIONS | 1.524364707 | 0.050073638 | 0.183603338 | 0.144950004 |

NES, Normalized Enrichment Score.
